#  Cofactors and Their Association with Cancer of the Uterine Cervix in Women Infected with High-Risk Human Papillomavirus in South India

**DOI:** 10.31557/APJCP.2019.20.11.3415

**Published:** 2019

**Authors:** Krishnan Baskaran, P Kranthi Kumar, K Santha, Inmozhi Sivakamasundari

**Affiliations:** 1 *Department of Biochemistry, Rajah Muthiah Medical College and Hospital, Annamalai University, Annamalainagar, Tamil Nadu, *; 2 *Department of Genetics, Narayana Medical College & Hospital, Nellore, Andhra Pradesh, India. *

**Keywords:** Human papilloma virus, cervical dysplasia, cervical cancer, cofactors

## Abstract

**Background:**

Human papilloma viruses (HPVs) are recognized as the major etiological agents of most pre invasive and invasive cancer of the uterine cervix. Many cofactors in association with high-risk HPV (HR-HPV) trigger infection which leads to cervical carcinogenesis. The aim was to study various cofactors and their association with cervical cancer in women infected with HR-HPV.

**Methods:**

The present study screened a total of 156 subjects for the presence of HPV infection. Association of various cofactors with cervical cancer was estimated using binary logistic regression analysis.

**Results:**

The HR-HPV infection showed a very significant risk factor for cervical cancer. Among the cofactors, the education level, early sexual exposure and age at pregnancy had no significant association while low socioeconomic status (SES) and high parity showed significant association as risk factors for cervical cancer. Tobacco chewing with betel quid was not significantly associated with cervical cancer.

**Conclusions:**

The present study indicates that low SES is a major risk factor associated with cervical cancer. Bringing awareness about HPV infection and intensifying routine screening programs for cervical cancer will help reduce the risk of cervical cancer among women with low SES in this region.

## Introduction

Cervical cancer is a malignant neoplastic disease of the uterine cervix. It is the fourth most common cancer affecting women worldwide with an estimated 570,000 new cases in 2018 (Bray et al., 2018). Mortality rates are considerably lower than morbidity. There were an estimated 311,000 deaths from cervical cancer worldwide in 2018 (Bray et al., 2018). About 70% of the global burden falls in developing countries and more than one fifth of all new cases are reported in India. For instance, in India, it is the second most common cancer among women with more than 96,922 new cases and 60,078 deaths occurring every year (Bruni et al., 2018). Cervical cancer is usually preceded by a long pre invasive phase, known as dysplasia. Cervical dysplasia is referred histologically as cervical intraepithelial neoplasia (CIN) or cytologically as squamous intraepithelial lesion (SIL). 

Human papilloma virus (HPV) is the major etiological agent of most pre invasive and invasive cancer of the uterine cervix. Infection and persistence of high-risk HPV (HR-HPV) are essential for the development of SIL and cervical cancer (Bosch et al., 2002; Muñoz et al., 2006). HR-HPV infections are common among sexually active young women. Most women clear the infection within one to two years and only some women develop SIL which subsequently progresses to cervical cancer (Bruni et al., 2010; Howell-Jones et al., 2012). Therefore, it implies that besides HR-HPV infection, many other factors might play a role as cofactors in the development of SIL and progression to cervical cancer (Muñoz et al., 2006; de Sanjosé et al., 2018). The various cofactors implicated with cervical cancer are low education, low socioeconomic status (SES), early age of marriage / early sexual exposure, high parity, multiple sexual partners, smoking/tobacco and oral contraceptive pills (OCPs) (Bassal et al., 2016; de Sanjosé et al., 2018). If their association as risk factors are unravelled, that knowledge could be utilized in the devising methods to prevent risk of cervical cancer. Therefore, the aim was to study various cofactors associated with cervical cancer in women infected with HR-HPV. 

## Materials and Methods

A total of 156 subjects in the age group of 30 to 65 years were screened for the presence of HPV infection by polymerase chain reaction (PCR) in outpatient clinic in the Department of Obstetrics and Gynecology, Rajah Muthiah Medical College and Hospital, Annamalai University, Annamalainagar, Tamil Nadu, India and in the Department of Obstetrics and Gynecology, Thanjavur Medical College, Thanjavur, Tamil Nadu, India. Inclusion criteria: Subjects in the age group of 30 to 65 years with no history of hysterectomy / conization / previous treatment of cervical cancer, and currently undergoing any treatment for cervical diseases and presently not pregnant were included. Exclusion criteria: Subjects with any serious and systemic illness like cardiac disease, any other malignancy and other sexually transmitted diseases were excluded. The study was approved by the Institutional Human Ethics Committee (RMMC, 2010) and the informed consent was obtained from each subject.

*Sample collection*

Cervical scrapings were collected using a sterile disposable cervical brush (Astra Scientific Systems Pvt. Ltd. Kerala, India) in a sample collection buffer [Phosphate buffer saline (PBS) pH 7.4] for the detection of HPV DNA. Biopsy specimens were obtained using a punch biopsy in neutral-buffered formalin [(NBF) (10%)] for histopathological studies. There were a total of 67 subjects with cervical cancer. They consisted of 65 squamous cell carcinoma (SCC) and two adenosquamous carcinoma (ADSC). There were a total of 61 subjects with SIL: 26 subjects with high-grade squamous intraepithelial lesion (HSIL) while 35 with low-grade squamous intraepithelial lesion (LSIL). 28 subjects, who were posted for hysterectomy with non-cervical diseases such as uterine prolapse, were chosen as controls. The study subjects were interviewed using a standardized questionnaire to get information on their education, economic status, marital status and parity, history of smoking / tobacco chewing, usages of OCPs and other mode of contraception. 

*Study population*

Association of various cofactors with cervical cancer was estimated by applying binary logistic regression analysis.In order to apply the binary logistic regression analysis, all LSIL (n=35) were added to controls (n=28) to make one group as controls (n=63) while all HSIL (n=26) were added to cervical cancer to make another group as cancer cases (cervical cancer) (n=93).

*Detection of HPV infection*

Detection of HPV infection with genotyping was performed by PCR (ApmliGenei HPV detection kit, Bangalore Genei, Bangalore, India). This kit detects eight HR-HPV types: HPV 16, 18, 31, 33, 35, 45 52 and 58. These sub types were detected based on the PCR product size varying between 230 and 270 bp (base pair). The details of PCR technique and the findings were given in the earlier publication (Baskaran et al., 2015). These eight HR-HPV types in the descending order of frequency are: HPV 16, 18, 45, 31, 33, 52, 58, 35 and they are responsible for about 90% of all cervical cancers worldwide (Munõz et al., 2006).

*Statistical analysis*

To find out the intensity of relationship between dependent (HPV infection) and independent variables (cofactors), binar (cofactors), binary logistic regression analysis y logistic regression analysis was applied. The Hosmer - Lemeshow test showed that the model was a good fit for data as the Chi-square value was not significant (p=0.351). The results were considered statistically significant if the p values of binary regression coefficient were equal or less than 0.05 (Table 3). The statistical analysis was carried out using Statistical Package for Social Science (SPSS) version 20 (IBM Corp, NY, USA).

**Table 1:** Detection of HPV Infection among Study Subjects

Study subjects	HPV positive (%)	HPV negative (%)
Controls	10.7 (3)	89.3 (25)
LSIL	65.7 (23)	34.3 (12)
HSIL	84.6 (22)	15.4 (4)
Cervical cancer	94 (63)	6 (4)

LSIL, Low-grade squamous intraepithelial lesion; HSIL, High-grade squamous intraepithelial lesion Data are expressed as percentage. Controls (n=28); LSIL (n=35); HSIL (n=26); Cervical cancer (n=67).

**Table 2:** HPV Status and Cofactors among Controls and Cancer Cases

Characteristics	Controls	Cancer cases
Average age in years	43	46
HPV status: Positive	41.3% (26)	91.4% (85)
Education		
Less than elementary	57.2% (36)	84.9% (79)
Elementary	33.3% (21)	9.7% (9)
High school	9.5% (6)	5.4% (5)
Economic status*		
Low	82.5% (52)	89.2% (83)
Middle	17.5% (11)	10.8% (10)
High	-	-
Sexual debut		
16 – 17 years	19.1% (12)	38.7% (36)
18 – 19 years	33.3% (21)	40.9% (38)
19 – 20 years and above	47.6% (30)	20.4% (19)
Age at pregnancy		
Before 20 years	49.2% (31)	67.7% (63)
After 20 years	50.8% (32)	32.3% (30)
Live birth / No. of FTP		
0 – 1	7.9% (5)	-
2 – 3	63.5% (40)	38.7% (36)
4 – 6	28.6% (18)	61.3% (57)
> 7	-	-
Tobacco with betel quid		
≥ 5 years	22.2% (14)	49.5% (46)
Duration of OCP usage		
0 years	100% (63)	100% (93)
Condom usage		
Never	98.4% (62)	100% (93)
Rarely	1.6% (1)	-
Frequently/always	-	-
Tubal ligation	69.8% (44)	63.4% (59)

FTP, Full-term pregnancy; OCP, Oral Contraceptive Pill; Economic status*: Based on National Planning commission, Government of India. Data are expressed as percentage. Control (n=63); Cancer cases (n=93).

**Table 3:** Regression Coefficients of HPV Status and Cofactors

Variables	B	S.E.	Wald	df	Sig.	Exp (B)	95% C.I.for EXP (B)	
							Lower	Upper
HPV status	2.932	0.703	17.403	1	0.001	18.767	4.733	74.416
Education	-0.897	0.63	2.028	1	0.154	0.408	0.119	1.402
Economic status	3.277	1.143	8.212	1	0.004	26.486	2.817	249.042
Sexual debut	0.251	0.279	0.813	1	0.367	1.286	0.744	2.221
Age at pregnancy	-0.393	0.225	3.064	1	0.08	0.675	0.435	1.048
Live birth / No. of FTP	0.479	0.225	4.534	1	0.033	1.615	1.039	2.511
Tobacco	-0.128	0.502	0.065	1	0.799	0.88	0.329	2.356
Constant	-0.575	4.427	0.017	1	0.897	0.563		

S.E., Standard error; C.I, Confidential interval; FTP, Full-term pregnancy

## Results

Table 1 shows prevalence of HPV infection among controls, LSIL, HSIL and cervical cancer. HPV DNA was detected in 10.7% (3) controls, 65.7% (23) LSIL, 84.6% (22) HSIL while it was detected in 94% (63) cervical cancer. Table 2 shows the association of education, economic status, early sexual debut / early consummation of marriage, age at pregnancy, parity [live birth / full-term pregnancy (FTP)], chewing tobacco (with betel quid) usages of OCPs, condoms and other mode of contraception between controls and cancer cases. The average age of controls was 43 years while it was 46 years for cancer cases. Among 63 controls, 26 (41.3%) were HPV positive while out of 93 cancer cases, 85 (91.4%) cancer cases were positive for HPV. The HPV infection showed a very significant risk factor for cervical cancer (p=0.001). Among the cofactors, the education level had no significant association as risk factor for cervical cancer (p=0.154). Low economic status revealed significant association as risk factor for cervical cancer (p=0.004). Early sexual debut or early sexual exposure had no significant association as risk factor for cervical cancer (p=0.367). Similarly, age at FTP whether it was before 20 or after 20 years showed no significant association as risk factor for cervical cancer (p=0.08). However, high parity (4-6 FTP) showed significant association as risk factor for cervical cancer (p=0.033). Tobacco chewing along with betel quid was not significantly associated with cervical cancer (p=0.799). The data on OCPs were not subjected to statistical analysis as none of the study subject used OCPs. Only one study subject reported using condom and tubal ligation was the mode of contraception in most cases. 

## Discussion

The HR-HPV infection showed a very significant risk factor for cervical cancer. Host immune system response plays a major role in HR-HPV clearing or progressing into cervical cancer. Understanding molecular mechanism leading to cervical cancer progression can enable devising strategies in cervical cancer mitigation. However, the present study focused on selected cofactors and showed low education was not significantly associated as risk factor for cervical cancer. Earlier studies reported that low education / illiteracy was observed as risk factor for cervical cancer in a case-control study in South India (Rajkumar et al., 2003; Franceschi et al., 2005). Education level as such cannot be taken as well defined exposure, but reflects the low SES (Franceschi et al., 2009). Low SES was shown to be a risk factor for cervical cancer (Franceschi et al., 2005; Singh et al., 2012; Alsbeih, 2015). The finding is in good agreement with these observations. Low SES as risk factor for cervical cancer can be attributed to lack of screening and awareness about HPV infection as well as cervical cancer (Tadesse, 2015). Moreover, lack of hygiene and poor nutrition might also play a contributing role as risk factors, which again reflect the low SES (Rajkumar et al., 2003; Thakur et al., 2015).

Early sexual exposure, age at FTP and number of FTP were associated with SIL and cervical cancer. Early sexual debut as risk factor could likely be due to early exposure, acquire and retention of HPV infection for longer duration (International Collaboration of Epidemiological Studies of Cervical Cancer, 2009; Jensen et al., 2013). However, the present study found early sexual debut and age at pregnancy were not shown to be risk factors for cervical cancer while high parity (live birth more than four) was shown to be a risk factor for cervical cancer. Risk of high parity with cervical cancer can be attributed to immunomodulation by increased levels of estrogens and progesterone and delivery-related cervical trauma which causes the eversion of the columnar epithelium onto the ectocervix which in turn leads to exposure to HPV infection. (Jensen et al., 2013; Thakur et al., 2015; Sun et al., 2017). Multiple sexual partners are shown to be the important risk factors for cervical cancer (Franceschi et al., 2005; Liu et al., 2015). However, most of the women in the present study were reported to be living with their husbands. 

Among various cofactors, tobacco smoking is shown to be one of the risk factors for cervical cancer (Haverkos et al., 2003; Wang et al., 2009; Xu et al., 2018). The study subjects were not smokers, but they had the habit of chewing tobacco with betel quid (A betel quid is a combination of betel leaf, areca nut and slaked lime). Chewing tobacco along with betel quid is common in Asia especially in India. The study subjects were chronic chewers of tobacco (more than five times per day and more than five years) with betel quid. The present study found tobacco chewing with betel quid was not significantly associated with cervical cancer. This is in contrast to previous study which showed significant association of chewing tobacco with betel quid among women with cervical cancer (Rajkumar et al., 2003). Tobacco chewing with or without betel quid is common among people with low SES. Therefore, it is probable that this association could be due to low SES (Gajalakshmi et al, 2012).

Prolonged use of OCPs is associated with an increased risk of cervical cancer in HR-HPV infected women (deVilliers et al., 2003; Roura et al., 2016; Xu et al., 2018). However, the association of OCPs with cervical cancer as risk factor could not be ascertained as none of the study subject used OCPs and most of the study subjects had tubal ligation as the major form of contraception in this region. Further studies among urban population with history of prolonged use of OCPs will throw more light on the status of OCPs in this region. Similarly, most of the study subjects never used condoms as a result the present study could not ascertain the association of condoms with HPV infection. However, earlier studies reported inconsistency about the effectiveness of condoms in preventing HPV infection (Raychaudhuri and Mandal, 2012; Roik et al., 2018). The present study has limitation because of its smaller sample size and therefore a study with a larger sample size is required to strengthen these findings. In conclusion, the present study reveals that low SES is a major risk factor associated with cervical cancer. Awareness programs about HPV infection and intensifying routine screening programs for cervical cancer will help reduce the risk of cervical cancer among women with low SES in this region.
